# Revisiting facial resemblance in couples

**DOI:** 10.1371/journal.pone.0191456

**Published:** 2018-01-18

**Authors:** Yetta Kwailing Wong, Wing Wah Wong, Kelvin F. H. Lui, Alan C.-N. Wong

**Affiliations:** 1 Department of Educational Psychology, Faculty of Education, The Chinese University of Hong Kong, Shatin, Hong Kong; 2 Department of Applied Social Sciences, City University of Hong Kong, Kowloon Tong, Hong Kong; 3 Department of Psychology, The Chinese University of Hong Kong, Shatin, Hong Kong; Macquarie University, AUSTRALIA

## Abstract

It is widely believed that couples look alike. Consistently, previous research reported higher facial similarity for couples than non-couples, and that facial similarity predicts marital satisfaction. However, it is unclear if facial similarity in couples shown in previous studies was solely driven by extrinsic features like hairstyle, glasses, etc. Also unclear is what attributes are perceived as similar from the faces of a couple. In three experiments, we showed that faces were considered more similar in couples than non-couples even without extrinsic features. Personality and age perceived from faces were also more similar in couples. Importantly, by matching pairs of faces according to their perceived personality, we found that a higher similarity in the perceived personality of a face pair led to higher facial similarity and couple likelihood ratings. These findings suggest that, instead of a result of pure physical analyses, facial similarity in couples is partly based on active social cognitive judgments on perceived personality, which may reveal the actual personality of the couples and thus inform relationship quality.

## Introduction

It is widely believed that couples look alike. In various cultures, similar-looking couples are often regarded as good matches. Consistent with this belief, previous research reported higher facial similarity for couples than non-couples, and that facial similarity predicts marital satisfaction [1–5]. This phenomenon has fascinated researchers debating on its underlying causes, ranging from mating for similarity in genetic composition for evolutionary benefits (e.g., in fitness and communication; [6]), seeking for a self-like partner as a form of narcissistic behavior [1], to forming similar facial features as a result of long-term exposure to facial musculature of the spouse’s expression [5].

A common assumption behind many theories lies in the importance of the stable, intrinsic features of a face in couple resemblance, e.g., the shape of the eyes, nose, mouth, and chin, that are separate from the more malleable, extrinsic features such as hairstyle, earrings, glasses, and make-up. For example, the genetic [6] and the long-term exposure accounts [5] are based on stable intrinsic features. Yet this assumption has not been robustly tested. All previous studies used face images that included not only the intrinsic facial features but also other malleable extrinsic features involving hairstyle, glasses, earrings, make-up, or even clothing [1–5]. In Zajonc et al. (1987) [5], for example, the effect of extrinsic features was controlled by judging faces only with hair style and facial outline. However, the influence of these cues could be smaller when the internal features were absent. Besides, other cues including eye gaze, glasses and posture still existed in the full-face condition. The observed facial similarity in couples could therefore be attributed largely to the similarity of these extrinsic features, which could be dramatically changed easily. If this were the case, then many theories of facial resemblance in couples would require modification or even become obsolete.

On the other hand, to understand why the facial similarity of two persons reveals their relationship quality, it is critical to understand *what* exactly is perceived as similar in the two faces, an issue that remains unexplored. Intuitively, similarity judgments may reflect the overall physical similarity of the whole faces, e.g., the shape, size and position of the features which is largely constrained by genes [1, 6]. It follows that any computerized algorithms that satisfactorily analyze the physical features of faces should result in human-like judgment on couple resemblance (although we are not aware of any direct empirical evidence testing this idea). This is consistent with the claims of some online-dating sites that computational analysis of overall physical similarity of two faces can help determine whether two individuals are a good match (e.g., ‘FindYourFaceMate’). Presumably, some of the physical features available on a photograph support perception of psychosocial qualities, such as age, attractiveness, and personality. Indeed these attributes have been shown to be perceived reliably and accurately from photographs of faces [7, 8]. Previous studies also showed that husbands and wives tend to be similar in these perceived attributes [4, 9, 10]. An intriguing question, therefore, concerns whether facial similarity reflects similarity of these perceived characteristics in couples.

In Experiment 1, we tested whether facial resemblance in couples is observed after removing all extrinsic cues from the facial images. Also we examined whether differences in perceived age, perceived attractiveness and perceived personality predict facial resemblance in couples. In Experiments 2, we focused on perceived personality and asked if manipulating the differences in the perceived personality of two faces would cause the faces to be considered more similar. Apart from facial similarity, couple resemblance has also been commonly measured by having naïve observers to judge the likelihood that the two individuals are couples [1–3, 5]. Experiment 3 tested whether the contribution of perceived personality to facial resemblance reveals specific information about couples. We asked if a pair of faces, matched with different levels of similarity in perceived personality, would be considered to belong to couples to various extents. Since facial similarity could be generally related to any relationship between two persons, such as friends or family members, it may not inform anything specific about couples, or as much as couple likelihood ratings would. Therefore we expected that the effect of perceived personality on couple resemblance measured by couple likelihood ratings would be equal to or even stronger than that measured by facial similarity.

## Experiment 1

The primary aim of this experiment is to test whether facial resemblance in couples would still be observed after all extrinsic cues from the facial images were removed. The differences in perceived age, perceived attractiveness and perceived personality were also measured to know if they would predict facial resemblance in couples.

### Materials and methods

#### Participants

Fifty-one university students (41 females and 10 males, *M* = 25.5 years, *SD* = 7.4, range = 18–46) were recruited as voluntary judges at City University of Hong Kong for course credit. The sample size was determined based on the predetermined rule that the experimenter would recruit as many participants as possible in two weeks of data collection, with a minimum number of participants matching the sample size in prior studies (ranging from 20 to 37; [4, 5]). The research was approved by the Ethics Review Committee of City University of Hong Kong, and informed consent was obtained in written form according to the committee’s guidelines.

#### Stimuli

**Fig 1:**
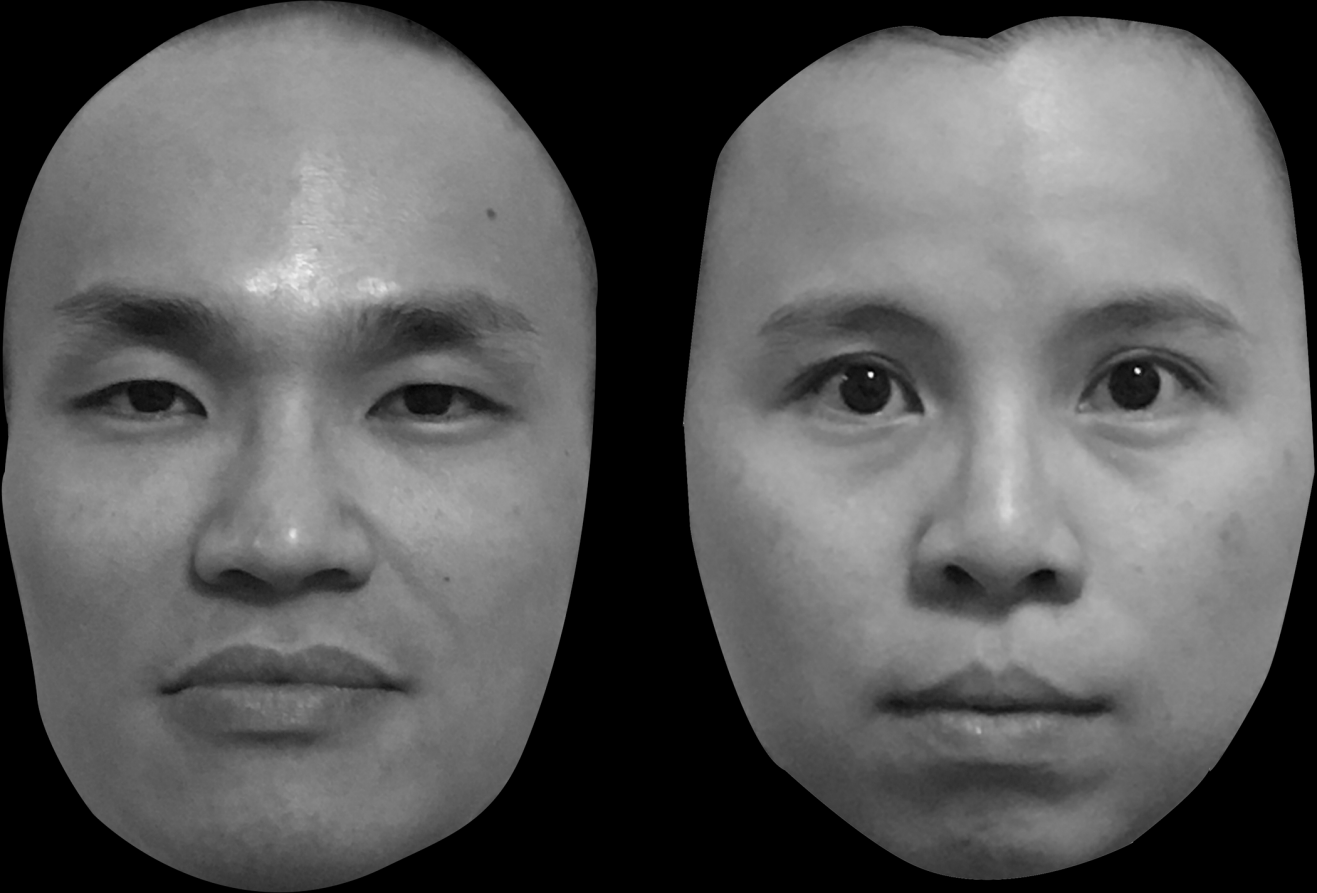
A sample pair of couple faces with only intrinsic facial features. Colored images of the faces were used in Experiment 1 and grayscale images were used in Experiments 2 and 3. These sample images were not used in the actual experiments. These two individuals have given written informed consent, as outlined in PLOS consent form, to publish these images.

Colored photographs of 60 pairs of married couples were taken with a neutral expression, relaxed facial muscles, and without glasses or makeup. Only intrinsic facial features were included, with the hair, ears, neck and shoulders removed (Fig 1). The couples were married from 0.5 to 35 years (*M* = 8.13 years, *SD* = 7.57). The wives and husbands were 26 to 55 years old (*M* = 36.6 years, *SD* = 5.84) and 28 to 60 years old (*M* = 38.3 years, *SD* = 6.39) respectively. Each face was presented at a visual angle of 5.8° × 8.2°, or 6.8 × 9.8cm with a viewing distance of about 68cm.

#### Procedure

The judges performed four types of rating on the photos of faces on a computer, including perceived facial similarity, perceived attractiveness, perceived personality and perceived age. First, they were presented pairs of faces side by side on a computer screen with the male face always on the left and the female face on the right. Judges rated the similarity of two faces on a 7-point Likert scale, where 1 represented faces that were extremely dissimilar, and 7 represented faces that were extremely similar, like brothers and sisters. The face pairs either belonged to real couples or non-couples that were created by shuffling the couple pairings. There were 120 trials in total.

Second, judges rated the perceived attractiveness of individual faces on a 7-point Likert scale, where 1 represented very unattractive faces and 7 represented very attractive faces. There were 120 trials in total.

Third, judges rated the perceived personality traits of individual faces according to the Five-Factor Model of Personality [11]. For each face, the five perceived traits were rated in a fixed order for consistency: openness to experience, neuroticism, extraversion, agreeableness and conscientiousness. For each trait, a 7-point bipolar semantic scale was used to represent the bipolar description of the traits (e.g., openness or closedness to experience, extraversion or introversion, agreeableness or antagonism, neuroticism or emotional stability, and conscientiousness or undirectedness), with the top five factor loadings of the traits provided on the questions to qualify the meaning of the traits and to ensure that participants were well-informed about the meaning of the traits [11]. Similarity of the overall perceived personality was summarized by the root-mean-square (RMS) of the face pair’s differences in the five traits with the equation:

$$RMS=\sqrt{\frac{{\left(M_{o,f}-M_{o,m}\right)}^{2}+{\left(M_{c,f}-M_{c,m}\right)}^{2}+{\left(M_{e,f}-M_{e,m}\right)}^{2}+{\left(M_{a,f}-M_{a,m}\right)}^{2}+{\left(M_{n,f}-M_{n,m}\right)}^{2}}{5}}$$ (1)

in which *M* stands for the mean ratings of all participants for each face, the subscripts of *o*, *c*, *e*, *a*, *n* stand for the five personality traits of openness, conscientiousness, extraversion, agreeableness and neuroticism respectively, and the subscripts of *m*, *f* stand for males and females respectively. RMS of the face pair’s differences were used with the following reasons. First, it has been well documented that various personality dimensions can be reliably and accurately perceived from photos of faces [4, 7], and we do not have strong theoretical reasons to stress more on some of the personality attributes than others in the search of influence of perceived personality on facial similarity. Second, limited by the number of couple pairs we had as face stimuli, we did not have the statistical power to treat each of the Big Five dimensions as individual factors in the multivariate model. Therefore we assumed equal importance of each personality dimension by taking the root-mean-square approach in this first step exploring the role of perceived personality in couple resemblance. There were 600 trials in total.

Finally, judges rated the perceived age of individual faces in decade (ranging from 20s to 80s) and then the position in that decade (early, mid and late). On the same screen, they also indicated if they knew the person and had any relationship with the person (e.g., friends, relatives, colleagues or classmates, if any). There were 360 trials in total (three questions for each face). For perceived attractiveness, personality and age, the individual faces were blocked by gender with the block order counterbalanced across judges. The order of the faces was randomized within each block. None of the judges reported knowing any of the rated persons. All questions were presented in both English and Chinese, with the translation to Chinese confirmed by back-translation. No time limit was imposed on the ratings. Each of the 120 faces was presented five times: once for facial similarity judgment as real couples, once for facial similarity judgment as non-couples, once for perceived attractiveness, once for perceived personality, and once for perceived age). There were 1200 trials in total for the whole experiment, and it lasted for around 1.5 hour for each participant.

### Results

Consistency was high among the judges’ ratings of similarity between face pairs (Cronbach’s *α* = 0.94), as well as perceived age (*α* = 0.99), perceived attractiveness (*α* = 0.97) and perceived personality traits (*α* = 0.81–0.93 for the five traits) of individual faces.

**Fig 2:**
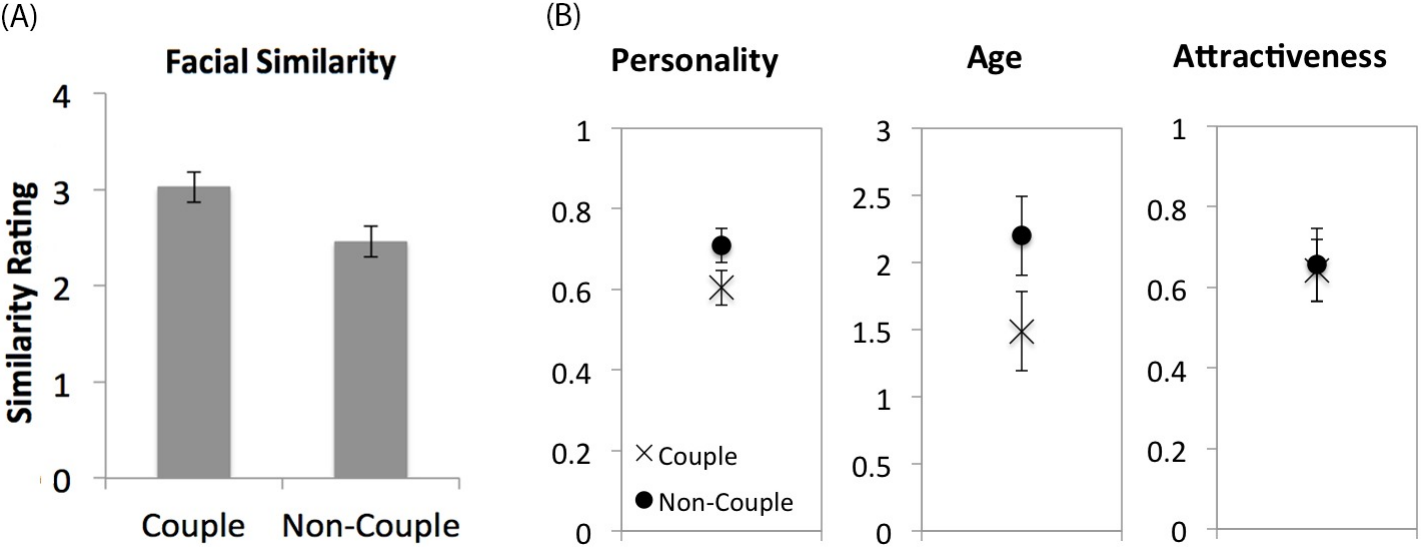
Results of Experiment 1. (A) Facial similarity in couples and non-couples rated by judges. The error bars represent the 95% confidence intervals for the difference between couple and non-couple faces. (B) Differences in perceived personality, perceived age and perceived attractiveness in face pairs of couples compared with that in non-couple pairs generated by bootstrapping. For perceived age, each unit represents 1/3 of a decade. The error bars represent the 95% confidence intervals of each condition.

An independent-samples *t*-test showed that facial similarity was higher for face images of couples (*M* = 3.03, *SD* = 0.65) than non-couples (*M* = 2.46, *SD* = 0.58), *t*(118) = 5.03, *p* < .001, Cohen’s *d* [95% CI] = 0.93 [0.54, 1.3] (Fig 2A). It demonstrated that, even when the external features are removed, faces of a couple look more similar than faces of a non-couple.

To test whether couples were more similar than non-couples in terms of perceived age, personality and attractiveness, bootstrapping analyses were conducted. The faces of a couple can be considered one specific pairing of faces among many possible pairings of the available faces. By bootstrapping, distributions of similarity of the perceived attributes between randomly paired faces were created based on the sample statistics, which allowed us to test theoretically how likely one can observe the values obtained in real couples in terms of a *p*-value. For each of these perceived attributes, a random distribution of the perceived difference of face pairs was generated by shuffling the couple pairing for 10000 times. The mean perceived difference of the 60 couple face pairs was then compared with the simulated random distribution to obtain the *p*-values as in two-tailed tests. It was found that couples were less different from each other than non-couples in both perceived personality (*M* = 0.604 vs. 0.709, *p* < .001, 95% CI = 0.086 [0.665, 0.751], range: [0.629–0.794], *SD* = 0.022) and perceived age (*M* = 1.49 vs. 2.20, *p* < .001, 95% CI = 0.588 [1.88, 2.47], range: [1.54–2.67], *SD* = 0.15), but did not differ in terms of perceived attractiveness (*M* = 0.64 vs. 0.66, *p* = .70, 95% CI = 0.153 [0.58, 0.73], range: [0.506–0.789], *SD* = 0.039; Fig 2B).

To test if differences in perceived age and perceived personality independently predicted facial resemblance, we computed the correlations between these variables, followed by a hierarchical regression analysis using both couple and non-couple face pairs. The correlation between facial resemblance and difference in perceived age was *r*(118) = -.207, *p* = .023, and that between facial resemblance and difference in perceived personality was *r*(118) =.-194, *p* = .034. In the hierarchical regression analysis, facial similarity was the dependent variable, difference in perceived age was the predictor entered in block 1, and difference in perceived personality was the predictor entered in block 2. Block 1 regression results showed that facial resemblance of couples was significantly contributed by perceived age difference (*R*^2^ = .043, *F*(1,118) = 5.30, *p* = .023). Block 2 regression results showed that the contribution of perceived personality difference over and above that of perceived age difference was marginally significant (Δ*R*^2^ = .030, Δ*F*(1,117) = 3.84, *p* = .052). Both perceived age difference (β = -.091, *SE* = .043, *t* = -2.13, *p* = .035) and perceived personality difference (β = -.390, *SE* = .199, *t* = -1.96, *p* = .052) contributed independently to perceived facial similarity.

### Discussion

In Experiment 1, we showed that facial resemblance was higher for couples than non-couples even when the extrinsic features were removed. Differences in perceived age and perceived personality were smaller for couples than non-couples, whereas perceived attractiveness did not differ between couples and non-couples. This seems inconsistent with previous report of positive assortative mating in physical appearance [4, 9, 10, 12]. However, it is consistent with a previous study that couples were not correlated in physical attractiveness when all extrinsic, stylistic cues were removed [13], suggesting that similarity in perceived attractiveness in couples may be largely based on extrinsic, stylistic cues.

More importantly, facial resemblance in couples was uniquely predicted by differences in perceived age and perceived personality. It is perhaps unsurprising and relatively trivial to observe the contribution of perceived age since couples tend to be more similar in age, and two young faces intuitively look more alike than a young face and an old face [14]. In contrast, it is interesting to observe that perceived personality still explained couple resemblance after controlling for the contribution of perceived age. An intriguing possibility is that facial similarity in couples is partly driven by their perceived personality.

## Experiment 2

To further examine the contribution of perceived similarity in personality on couple resemblance, we manipulated the difference in perceived personality by selecting different pairs of faces and observing for each pair the perceived facial resemblance. Observers rated the similarity between a target face and the face of (i) the spouse, (ii) another opposite-sex individual with a better personality match than the spouse, and (iii) another opposite-sex individual with a worse personality match than the spouse. If perceived personality determines, at least partly, facial resemblance, facial resemblance should be higher in face pairs with better-matched personality than worse-matched personality.

### Materials and methods

#### Participants

Sixty participants including 26 males and 34 females (*M* = 22.62, *SD* = 5.62, range = 18–52) were recruited as judges at the Chinese University of Hong Kong for monetary compensation. We aimed to have a comparable sample size to that in Experiment 1, and allowed sixty participants to sign up for the experiment in case of no-shows. The research was approved by the Survey and Behavioral Research Ethics Committee of the Chinese University of Hong Kong, and informed consent was obtained in written form according to the committee’s guidelines.

#### Stimuli

Using the face stimuli and ratings of Experiment 1, we paired each of the faces up with three types of faces: (i) the face of the spouse (the ‘couple pairs’); (ii) the face of another opposite-sex individual with a better personality match than the spouse (the ‘better-matched pairs’); and (iii) the face of another opposite-sex individual with a worse personality match than the spouse (the ‘worse-matched pairs’). In order to manipulate differences in perceived personality while having the differences in perceived age and perceived attractiveness controlled, the better- and worse-matched pairs were formed under three constraints. First, the difference in perceived personality (in RMS; data from Experiment 1) should be numerically smaller in the better-matched pairs than in the couple pairs, and larger in the worse-matched pairs than in the couple pairs. Second, the difference in perceived age should be comparable, i.e., less than 1/3 of a decade, between the couple pairs and the better-matched pairs, and between the couple pairs and the worse-matched pairs. Third, the difference in perceived attractiveness should be comparable, i.e., less than 0.5-point difference out of the 7-point scale, between the couple pairs and the better-matched pairs, and between the couple pairs and the worse-matched pairs. For some of the target faces, it was impossible to form the three face pairs under the above constraints. For example, in some cases the face of the spouse was also the face with the best personality match, while in some other cases all the faces with a better personality match than the couple pair did not have comparable differences in perceived age or attractiveness. As a result, out of the 120 faces (60 males and 60 females), 38 faces (18 male, 20 female) were used to produce 114 face pairs (3 face pairs for each face) as the stimuli. Confirming the manipulations, independent-sample *t*-tests showed that the difference in perceived personality was smaller for the better-matched pairs (*M* = 0.3237, *SD* = 0.1750) than the couple pairs (*M* = 0.5967, *SD* = 0.3018), *t*(62) = -4.54, *p* < .001, Cohen’s *d* [95% CI] = 1.14 [0.6, 1.69], and also smaller for the couple pairs than the worse-matched pairs (*M* = 1.1244, *SD* = 0.2742), *t*(64) = -7.40, *p* < .001, Cohen’s *d* [95% CI] = 1.84 [1.25, 2.44]. The differences in perceived age and in perceived attractiveness were comparable between the three types of face pairs (*ts* < 1.22, *p*s > .226 for all pairwise comparisons). Out of the 114 face pairs, 12 of them were repeated and so each participant only needed to rate the similarity of 102 pairs of stimuli. Each face was presented at a visual angle of 3.5° x 5.1°, or 4.2 x 6.1 cm with a viewing distance of 68 cm. The faces were changed to a grayscale version in order to minimize any potential effects of lighting or pigmentation on similarity ratings, such as those caused by taking the pictures in dim yellow versus bright white lighting. After changing the faces into grayscale images, while the faces still appeared to be lighter or darker as in individual differences in skin color, it became impossible to match the faces based on the color of the lighting and the effects of lighting or pigmentation on similarity ratings were thus reduced.

#### Procedure

In each trial, a pair of faces was presented side by side on a computer screen with the male face always on the left and the female face on the right. Judges rated the facial similarity of the 102 face pairs in a randomized order on a 7-point Likert scale, where 1 represented faces that were extremely dissimilar, and 7 represented faces that were extremely similar, like brothers and sisters. All questions were presented in both English and Chinese. Judges input their ratings on desktop computers without time limit.

### Results

**Fig 3:**
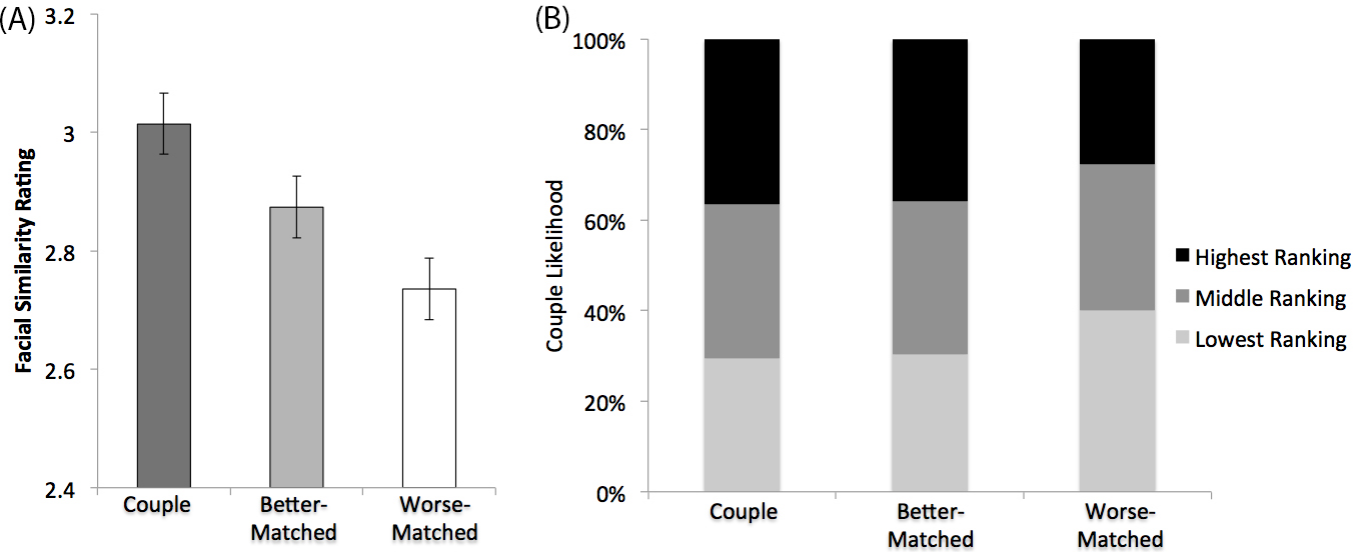
Results of Experiments 2 and 3. (A) Ratings of facial similarity in Experiment 2. The error bars represent the 95% confidence intervals of the main effect of face type. (B) Percentages of the highest, middle and lowest ranking in terms of the likelihood of belonging to a couple for the three types of face pairs in Experiment 3.

Consistency was high among the judges’ ratings of similarity between face pairs (Cronbach’s α = 0.908). Fig 3A shows the ratings of facial similarity for the three types of face pairs. A one-way repeated measure analysis of variance (ANOVA) showed a significant effect of face type, *F*(2,58) = 31.773, *p* < .001, *η*_p_^2^ [95% CI] = .523 [.33, .64] [15]. Post-hoc comparisons showed that facial similarity for the couple pairs (*M* = 3.01, *SD* = .844) was higher than that for the better-matched pairs (*M* = 2.87, *SD* = .829), *t*(59) = 3.75, *p* < .001, Cohen’s *d* [95% CI] = 0.48 [0.21, 0.75] and that for the worse-matched pairs (*M* = 2.74, *SD* = .788), *t*(59) =, *p* < .001, Cohen’s *d* [95% CI] = 1.04 [0.72, 1.35]. Facial similarity was also higher for the better-matched pairs than for the worse-matched pairs, *t*(59) = 3.63, *p* = .001, *d* [95% CI] = 0.48 [0.20, 0.73].

### Discussion

In Experiment 2, we found that face pairs were considered more similar when they were better matched in perceived personality, indicating that perceived personality contributes to the facial resemblance of couples. This cannot be explained by the differences in perceived age and perceived attractiveness since they were comparable between the three types of face pairs.

However, the facial similarity of couples was still higher than that of the best-matched couples, even though the best-matched couples had a smaller difference in perceived personality than couples. One possible reason is that the Big Five dimensions we used did not capture completely all of the critical personality features affecting facial similarity in couples. Another possibility is that facial similarity may be influenced by factors other than perceived personality, such as similarity in physical fitness, ethnicity, etc., that can be perceived in a face [16, 17].

## Experiment 3

Experiment 3 tested whether the contribution of perceived personality would also be found with couple likelihood, another common dependent measure adopted in the study of facial resemblance in couples [1–3, 5]. Observers ranked the likelihood to be couples for three types of face pairs–the couple pairs, the pairs better-matched in perceived personality, and the pairs worse-matched in perceived personality. If perceived personality determines which face pairs are likely to be considered couples, the better-matched pairs should be ranked higher in couple likelihood than the worse-matched pairs.

### Materials and methods

#### Participants

Sixty participants including 17 males and 43 females (*M* = 21.63, *SD* = 4.63, range = 18–45) were recruited as judges at the Chinese University of Hong Kong for monetary compensation. The sample size was determined based on that in Experiment 2. Informed consent was obtained in written form according to the guideline of the Survey and Behavioral Research Ethics Committee of the Chinese University of Hong Kong.

#### Stimuli and procedures

The same 114 pairs of faces generated in Experiment 2 were used, which contributed to three types of face pairs for each reference person: the couple pairs, the better-matched pairs and the worse-matched pairs in perceived personality. On each trial, the three face pairs for each reference person were arranged vertically, with the reference person’s face always located on the left and the other face in each face pair on the right. Each photograph was presented at a visual angle of 3.5° x 5.1°, or 4.2 x 6.1 cm with a viewing distance of 68 cm such that all of the three face pairs can be viewed on the screen simultaneously without the need to scroll up and down the page. Judges were asked to rank the three pairs of faces based on their likelihoods to be a couple, with 1 being the most likely and 3 being the least likely. The experiment was separated into two blocks according to the gender of the reference person’s face, with the male block presented before the female block for all judges. The photographs of the reference faces were randomized within each block and the positions of the three pairs of faces were randomized within each trial. All questions were presented in both English and Chinese.

### Results

Fig 3B shows the percentages of the highest, middle and lowest ranking in the likelihood of belonging to a couple for different face pairs. A chi-square test showed that the distribution of ranking differed between the three types of face pairs, *χ*^2^(4, *N* = 6840) = 79.90, *p* < .001, *φ*_*c*_ = .076. The couple pairs and the better-matched pairs were judged as more likely to be couples than the worse-matched pairs in perceived personality.

To further test if there was any difference in the couple likelihood ranking between the couple pairs and the better-matched pairs, another chi-square test with only couple and better-matched pairs was performed. Results showed no difference between their ranking frequency distributions, *χ*^2^(2, *N* = 4569) = 0.427, *p* > .250, *φ*_*c*_ = .01, indicating that the face pairs with better personality match were judged to be as likely to be couples than pairs of couple faces.

Results from Experiment 3 were consistent with those in Experiment 2 in that face pairs better matched in perceived personality were judged to be more likely to belong to couples than those worse matched (Experiment 3). However, findings diverged in these two experiments between the better-matched pairs and the couple pairs. Whereas the better-matched pairs were rated less similar than the couple pairs in Experiment 2, the better-matched pairs and the couple pairs were regarded as equally likely to belong to couples in Experiment 3.

One potential reason for the difference lies in the different data formats, i.e., similarity ratings in Experiment 2 and rankings in Experiment 3. It is possible that similarity ratings provide data with higher precision than ranking data, which makes it possible to differentiate between the couple pairs and better-matched pairs in Experiment 2 but not in Experiment 3. To test if this explains the difference, we recoded the similarity ratings in Experiment 2 into rankings 1 to 3 by comparing the similarity ratings of the three pairs of faces for the same reference face for each participant. A chi-square test showed that the ranking differed between the three types of face pairs, *χ*^2^(4, *N* = 6840) = 63.32, *p* < .001, *φ*_*c*_ = .068. The couple pairs and the better-matched pairs were judged to be more similar than the worse-matched pairs. The chi-square test with only the couple and better-matched pairs showed that the couple pairs were judged to be more similar than the better-matched pairs in perceived personality, *χ*^2^(2, *N* = 4560) = 19.65, *p* < .001, *φ*_*c*_ = .066. In sum, the pattern of results in Experiment 2 was similar regardless of whether the data format was ratings or rankings.

To further confirm whether judgment of facial similarity and couple likelihood produced different result patterns, we directly compared the ranking results in Experiment 2 and 3 with a loglinear model. The likelihood ratio test showed that the 2×3×3 interaction (Experiment × Types of face pairs × Ranking levels) was significant, *χ*^2^(4, *N* = 13680) = 11.597, *p* = .021. This confirms that the judgment of facial similarity and couple likelihood were different given identical stimuli, manipulated conditions and sample sizes.

### Discussion

In Experiment 3, face pairs were considered more likely to belong to couples when they were better matched in perceived personality. This was consistent with the findings in Experiment 2 in that perceived personality affects couple resemblance.

In the literature, similarity rating of faces and the likelihood of being couples are often adopted as dependent measures [1–3, 5], but their potential differences have not been systematically compared. In Experiment 2 and 3, the two dependent measures led to significantly different results with the same stimulus set, manipulated conditions and sample size. Specifically, when facial similarity rating was used, couple faces were considered more similar than faces better matched in perceived personality, but the couple likelihood measure showed no difference in these two conditions. It cannot be explained by the potential difference in a nominal ranking measure versus an ordinal measure of similarity rating, because recoding the similarity rating data into ranking did not eliminate the observed differences. One possibility is that these two types of judgment may affect the weighting of perceived information from the faces. Using similarity rating in Experiment 2 might have caused participants to emphasize on overall physical facial features. Using couple likelihood rating in Experiment 3, in contrast, may have caused participants to focus on facial features more relevant to the compatibility of two persons in a romantic relationship. These facial features may overlap more with those features supporting perceived personality similarity. This may have led to couples and better-matched faces considered equally likely to be couples in Experiment 3. One may want to take into account these potential differences in interpreting previous findings before this is resolved in future, as to our knowledge no studies have systematically examined differences between judgment of facial similarity and couple likelihood.

## General discussion

In three experiments, we asked whether facial resemblance in couples can be observed with only intrinsic facial features, and what underlies these similarity judgments. Experiment 1 demonstrated that faces were considered more similar in couples than non-couples even after extrinsic features were removed. Also, couples were more similar than non-couples in perceived personality and perceived age, and both factors independently and linearly predicted facial similarity in couples. Using experimental approaches, face pairs with better-matched perceived personality were considered more similar (Experiment 2), and more likely to belong to couples (Experiment 3), than face pairs with worse-matched perceived personality.

These findings advance our understanding of facial resemblance in couples in several aspects. First, we excluded a highly feasible possibility that couple resemblance is only a result of malleable external facial features, which would be in conflict with existing theories, including the genetic contribution to the facial resemblance [6] and the fine-tuning of facial features through long-term exposure [5]. For the narcissistic account of couple resemblance [1], both intrinsic and extrinsic features may contribute to the goal of looking for a similar-looking spouse. Future work on the relative importance of intrinsic and extrinsic features could further clarify the explanatory power of different accounts.

In addition, our findings offer new insights into why facial similarity informs relationship quality of a couple. When we judge facial similarity, we also read into and compare personality and age of the persons. This involves active social cognitive processes well beyond pure visual analyses of the faces. In everyday life, we attribute and make decisions concerning other individuals by extracting personality information from their faces, such as whether they are approachable, aggressive, neurotic, etc. [7]. Reading personality from faces is reliable across individuals and cultures, and are valid even when they are based on highly limited information, e.g., photos of strangers’ faces with a neutral expression [7, 8]. In other words, we can accurately infer a stranger’s actual personality by looking at their photos. Importantly, actual personality predicts marital satisfaction [18, 19]. For example, both husbands and wives reported higher marital satisfaction when their partner is more agreeable, conscientious, open and emotionally stable (or low in neuroticism; [18]). Given these links, it may not be surprising that overall facial similarity may indirectly infer relationship quality through the following cognitive processes: When judging facial similarity of two persons, one also actively extracts personality information from their faces, which informs the actual personality of the two persons, and indirectly predicts their relationship quality. While it is conceivable that predicting relationship quality from facial similarity may induce error under specific conditions, e.g., two highly neurotic persons may have similar faces but low relationship quality [18, 19], facial similarity may still be sufficiently informative in general about relationship quality such that this phenomenon is well received by the public in different cultures. Future studies should try to dissociate the specific personality attributes through which perceived similarity enhances couple resemblance, and those attributes with which perceived similarity has no effect on or even reduces couple resemblance.

The current results on couple resemblance were observed based on static face stimuli with a neutral expression and no extrinsic features, which appears to divert significantly from our daily experience. It is conceivable that effect of perceived personality and age on couple resemblance may interact with additional factors in real life scenario. Further work may examine, for example, the extent to which judgments of facial resemblance depend on extrinsic features (e.g., couples wearing hats and glasses of similar or different styles) and facial expressions (e.g., big smiles or stern expressions), and how much facial resemblance based on intrinsic vs. extrinsic features predict relationship quality.
